# Identification, Pathogenicity, and Antimicrobial Resistance Analysis of Bacterial Pathogenesis *Aeromonas hydrophila* from Hybrid Sturgeon (*Huso dauricus* ♀ × *A. schrenckii* ♂) in Zhejiang, China

**DOI:** 10.3390/microorganisms13020278

**Published:** 2025-01-26

**Authors:** Haojie Hu, Xinzhi Weng, Gang Pang, Xiaobing Li, Jing Xia, Xiu Gao, Jie He, Ji Li, Dong Qian

**Affiliations:** 1 School of Oceanography, Shanghai Jiao Tong University, Shanghai 200030, China; 2 Taizhou Extention Station for Fishery Technique, Taizhou 315800, China; 3 School of Marine Sciences, Ningbo University, Ningbo 315211, China; 4 Shanghai Institute of Immunity and Infection of the Chinese Academy of Sciences, Shanghai 201203, China; 5 University of Chinese Academy of Sciences, Beijing 100049, China

**Keywords:** hybrid sturgeon, *Aeromonas hydrophila*, identification, pathogenicity, antimicrobial resistance

## Abstract

In 2019, a disease outbreak struck a hybrid sturgeon farm (*Huso dauricus* ♀ × *A. schrenckii* ♂) in Tiantai, Zhejiang province, leading to the deaths of 8000 sturgeons. The sturgeons exhibited reduced appetite, lethargic and uncoordinated swimming, and physical signs such as reddish petechiae and ulcers on the body and fins. Hemorrhagic spots were observed on the kidneys, spleen, and gonads, alongside reddish intestines with hemorrhagic ascites in the abdominal cavity. ST-1902 was isolated and identified as *Aeromonas hydrophila* through physiological and biochemical characterization and 16S rDNA sequence analysis. The pathogenicity of ST-1902 was confirmed through a challenge test, with a median lethal dosage (LD50) of 7.9 × 10^6^ CFU/IND. Histopathological examination showed hyperplasia and neoplasm-like changes in the epicedial mesothelial tissues, enlarged and necrosis renal tissue, and serious hemosiderosis in spleen and gills. Virulent genes (*Aer*, *Epa*, *Alt*, *Hly*, and *Act*) were detected in ST-1902, corresponding to typical β-hemolysis, extracellular protease, and enterotoxin. Moreover, antimicrobial experiment detection indicated ST-1902 is sensitive to quinolones and phenicols but resistant to sulfamethoxazole, aminoglycoside antibiotics with *Sul1*, and *Intl* and *Ant* (*3”*)-I. These results suggest that *A. hydrophila* was the causative agent of the sturgeon disease and highlight the emerging threat it poses to the sturgeon industry.

## 1. Introduction

Sturgeon, an archaic fish species belonging to the Acipenseridae family, predominantly inhabits the temperate aquatic realms of the Northern Hemisphere. There are 27 recorded species of sturgeon, all of which are endangered and listed as CITES [1]. As the demand for caviar continues to rise, the sturgeon industry has undergone rapid global expansion over the past two decades, notably in China, Russia, Germany, the United States, and other countries [2,3]. China has been engaged in sturgeon farming since the Tang Dynasty in the seventh century and is now the largest producer of sturgeon, with a total production of 96,914 tons in 2018, an increase of 13,856 tons from 2017 [4]. The Chinese sturgeon (*A.sinensis*), Siberian sturgeon (*A. baerii*), Japanese sturgeon (*A. schrenckii*), Russian sturgeon (*A. gueldenstaedtii*), and Ur Kaluga (*Huso dauricus*) are important species around the world. Recently, the large hybrid (*Huso dauricus* ♀ × *A. schrenckii* ♂) and small hybrid (*A. schrenckii* ♀ × *Huso dauricus* ♂) have emerged as the primary cultivation focus owing to their exceptional growth rates and robust disease resilience [5,6].

The rapid expansion of intensive sturgeon farming has been accompanied by a surge in bacterial disease outbreaks [7], leading to high mortality rates and significant economic losses in the sturgeon industry. Bacterial infections are now considered the most serious diseases and the primary threat to the sturgeon industry [8]. Various bacterial species have been implicated in causing diseases in cultured sturgeons. *Aeromonas hydrophila* [9,10,11], *A. punctata* subsp *caviae* [12], *A. sobria* [13], and *A. veronii* [14] have been reported as causative agents of cultured sturgeon diseases. Additionally, *Streptococcus* [15], *Citrobacter freundii* [16], *Pseudomonas alcaligenes* [17], *Yersinia ruckeri* [18], and *Mycobacterium* [19] have been identified as bacterial pathogens associated with high mortality rates. Among these pathogens, motile aeromonads, which are ubiquitous in aquaculture settings, are the most frequently reported bacteria affecting sturgeon culture, resulting in substantial economic losses [20,21].

In 2019, a serious disease outbreak occurred in sturgeon farms in Tiantai, Zhejiang Province, eastern China, from July to September. Infected sturgeons displayed reduced appetite and sluggish swimming behavior. Anatomical examination revealed lesions in the kidneys, spleen, gonads, and liver, along with congested intestines and a heart with a mulberry-like appearance. Predominant bacteria isolated from the moribund sturgeon were identified as *A. hydrophila* through physiological and biochemical characterization, followed by 16S rDNA sequencing. The pathogenicity, main virulence genes, and antimicrobial drug resistance of isolate ST-1902 were also sequenced and analyzed.

## 2. Materials and Methods

### 2.1. Bacterial Isolation

Diseased hybrid sturgeons were kept in oxygenated bags and transported to the laboratory for bacterial isolation. The surface of the sturgeon was disinfected with a 75% alcohol spray before dissection. Samples from the liver, spleen, kidney, and heart of the moribund sturgeons were streaked with sterile swabs onto Nutrient Agar (NA) (Hopebio, Qingdao, China). The NA plates were incubated at 28 °C for 24 to 48 h. Single colonies were selected for further purification. Strain ST-1902 and thirteen other colonies (ST-1901, ST-1903 to ST-1914) were chosen for further culture and re-isolation. All isolated strains were stored at −80 °C in nutrient broth (Hopebio, Qingdao, China) with 15% (*v*/*v*) sterile glycerol. ST-1902, isolated from sturgeon with typical lesions, was selected for challenge tests, followed by physiological and biochemical characterization, as well as analysis of virulence and antibiotic resistance genes.

### 2.2. Challenge Test

Healthy hybrid sturgeons (*Huso dauricus* ♀ × *A. schrenckii* ♂), ranging from 25 to 35 mm in length and weighing 80 to 120 g, were obtained from Hangzhou Qiandaohu Lake Sturgeon Science and Technology Co. Ltd. (Hangzhou, China). The hybrid sturgeons, sourced from ponds with no disease records, were cultivated in an aeration aquarium and fed commercial pellet feed (Tianbang, Ningbo, China) at 28 °C for 7 days prior to challenge tests. The healthy sturgeons were then divided into six groups: five for the challenge tests and one for the control, with ten sturgeons in each group. ST-1902, the isolated and selected strain for the challenge test, was cultured in nutrient broth at 28 °C in an oscillating incubator for 18 h. The cultures were harvested by centrifugation at 4000 rpm for 10 min at 4 °C (Thermo, Waltham, MA, USA, ST16-R). The harvested culture was resuspended in autoclaved 0.9% saline, and the bacterial concentration was determined using a turbidimeter.

Fish were injected with bacterial concentrations of 1.8 × 10^9^, 1.8 × 10^8^, 1.8 × 10^7^, 1.8 × 10^6^, and 1.8 × 10^5^ CFU per fish in 0.2 milliliters. The control group was injected with an equivalent volume of saline. All fish were housed in six 0.075 m^3^ aquariums at 28 °C for 2 weeks. Mortality was recorded daily, and affected sturgeons were used for bacterial isolation on Nutrient Agar (NA) followed by identification using 16S rDNA sequencing.

The healthy sturgeons used in the challenge experiment and the research plan were reviewed and approved by the Ethics Committee of Ningbo University (No. SYXK20190005).

### 2.3. Physiological and Biochemical Characterization of Bacteria Isolates

ST-1902 was subjected to Gram staining according to the protocol of the Gram staining kit (Solarbio, Beijing, China). Bacterial identification was performed using the API 20E strip (Biomerieux, Craponne, France) with an ST-1902 suspension in 0.9% saline, incubated at 37 °C for 24 to 48 h. The results for ST-1902 were recorded at 24 h and 48 h, and identification was completed according to the instructions provided with the API 20E strip. The hemolysin and extracellular protease activities of strain ST-1902 were analyzed by incubating on 10% sheep blood agar and skim milk agar at 28 °C for 18 to 24 h.

### 2.4. Molecular Identification

The bacterial strains ST-1902 and thirteen others were cultivated in nutrient broth for 18–24 h prior to the extraction of total genomic DNA. The universal primers (27F 5′-AGAGTTTGATCCTGGCTCAG-3′/1492R 5′-GGCTACCTTGTTACGTTT-3′) of 16S rDNA were used for PCR amplification in 50 μL PCR systems added with 25 μL of 2 × Taq PCR Master Mix (TaKaRa, Dalian, China), 20 μL of deionized water, 40 pmol of each primer, and 3 μL of template DNA from ST-1902. The PCR reaction conditions were as follows: denaturation at 94 °C for 5 min; and 30 cycles of denaturation at 94 °C for 30 s, annealing at 55 °C for 1 min, extension at 72 °C for 90 s, plus a fiat extension step at 72 °C for 10 min. Amplified products were validated via 1.5% agarose gel electrophoresis and then confirmed by 1.5% agarose gel electrophoresis and sent to BGI for sequencing. The obtained sequences were compared by the Basic Local Alignment Search Tool (BLAST) of the National Center for Biotechnology Information (NCBI, https://www.ncbi.nlm.nih.gov/, accessed on 24 December 2024) database to identify the bacterial strains. To further identify species based on sequences, the 16S rDNA sequences of *Aeromonas*, *Yersinia*, *Edwardsiella*s, *Pseudomonas*, and *Streptococcus* strains (*Aeromonas sobria*, *Aeromonas hydrophila*, *Aeromonas media*, *Aeromonas veronii*, *Aeromonas encheleia*, *Aeromonas caviae*, *Aeromonas schubertii*, *Yersinia ruckeri*, *Edwardsiella ictaluri*, *Pseudomonas agarici*, *Streptococcus agalactiae,* and *Streptococcus iniae*) were downloaded from the NCBI database for comparisons. A phylogenetic tree was constructed with the Neighboring-Joining (NJ) method using MEGA 11 software with 1000 bootstrap replicates. The phylogenetic tree was optimized using the iTOL database (https://itol.embl.de/, accessed on 24 December 2024). After establishing the phylogenetic tree, the 16S rDNA sequences were uploaded to NCBI, and GenBank accession numbers were obtained subsequently.

### 2.5. Virulence Genes Analysis for ST-1902

PCR was used for detecting the putative virulence genes (Table 1) according to the isolated strains. The DNA template extracted with the boiling method was applied for the PCR system with reaction conditions according to the references [22,23]. The PCR products were confirmed by 1.5% agarose gel electrophoresis and sent for sequencing.

### 2.6. Histopathology Observation

The heart, liver, spleen, and kidneys of infected sturgeon were fixed in 10% neutral formalin for 24 h and subsequently replaced with fresh 10% neutral formalin for an additional 24 h. The tissues were dehydrated in a series of alcohol solutions, embedded in paraffin wax, and sectioned into 5 μm thick slices. These sections were stained with hematoxylin–eosin (H&E) and examined using an optical microscope for pathological analysis.

### 2.7. Antimicrobial Sensitivity Tests

The antimicrobial susceptibility of the isolate was measured using the Kirby–Bauer disk diffusion method with Mueller–Hinton (MH) agar (Solarbio, Beijing, China), in accordance with the standards set by the Clinical and Laboratory Standards Institute [24]. Eighteen antimicrobial agents belonging to the classes of penicillins, tetracyclines, quinolones, macrolides, and aminoglycosides were selected for the antibiotic sensitivity test of the isolate ST-1902. The inhibition diameter was measured after 24–48 h of incubation at 28 °C. An inhibition zone greater than 19 mm was recorded as susceptible, moderately susceptible for an inhibition zone ranging from 13 mm to 19 mm, and resistant for a zone smaller than 13 mm.

### 2.8. Antimicrobial Drug Resistance Gene Analysis

The specific primers (Table 1) of antimicrobial resistance genes, which had been reported in *A. hydrophila*, including sulfonamide (*sul1*), I-integron (*Intl*), transmethylase *Cfr*, aminoglycosides *Ant* (3″)-I, and florfenicol *FexA*, were synthesized by BGI. The DNA template was extracted by the boiling method, and the PCR system and reaction conditions followed the reference protocol. The PCR products were visualized and confirmed by 1.5% agarose gel electrophoresis [25,26,27].

## 3. Results

### 3.1. Symptoms of Diseased Sturgeon

**Figure 1:**
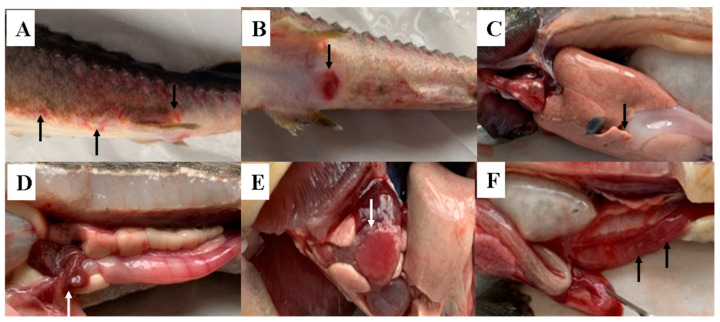
Signs of naturally infected sturgeon (black and white arrow showed). (**A**): Ulcers on sturgeon body surface; (**B**): swollen and hemorrhagic anus; (**C**): pale and enlarged liver; (**D**): hyperemic and enlarged spleen; (**E**): mulberry-like heart with vesiculated edge; (**F**): swollen intestine with hemorrhagic ascites.

**Figure A1:**
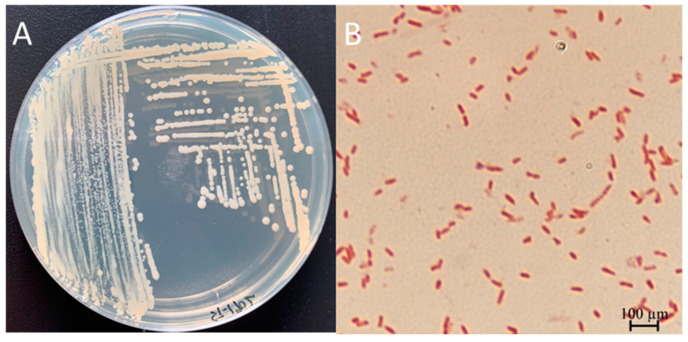
Bacterial colony (**A**) and Gram staining (**B**, 100×) of the isolate ST-1902.

In the first week of July 2019, a disease outbreak affected hybrid sturgeons being cultured in two cement tanks, (140 m^2^ each), each housing 20,000 juveniles (143 IND/m^2^). By the end of September, about 8000 dead sturgeons were recorded in each tank, with an average mortality rate of 40%. The diseased sturgeons exhibited decreased appetite and vitality, and swam around the pond. The common symptoms of the diseased sturgeons included the presence of reddish petechiae, ulcers on the body surface, fin rot, and visceral hemorrhage, particularly in the kidneys and spleen (Figure 1A). Some diseased fish had red and swollen anus (Figure 1B), red intestines, severe bleeding, and light-red hemorrhagic ascites (Figure 1D,F). The liver was pale and small punctate hemorrhages were visible, which may be caused by hemorrhage (Figure 1C). Hemorrhages were also present in the swollen spleen and gonad (Figure 1D). Grain-like cystic neoplasms were found in the heart, resembling the early mulberry-like heart as reported previously (Figure 1E). ST-1902 and thirteen other strains were successfully isolated from diseased sturgeons cultivated in the same farm through three isolations (Table A1). The isolated bacteria formed round, raised, smooth, and creamy colonies after culturing on NA at 28 °C for 24 h (Figure A1A). The bacteria were confirmed as Gram-negative, short rods with blunt, rounded ends, exhibiting active motility when observed under a phase-contrast microscope (Figure A1B).

### 3.2. The Pathogenicity of ST-1902 to Hybrid Sturgeon

**Figure 2:**
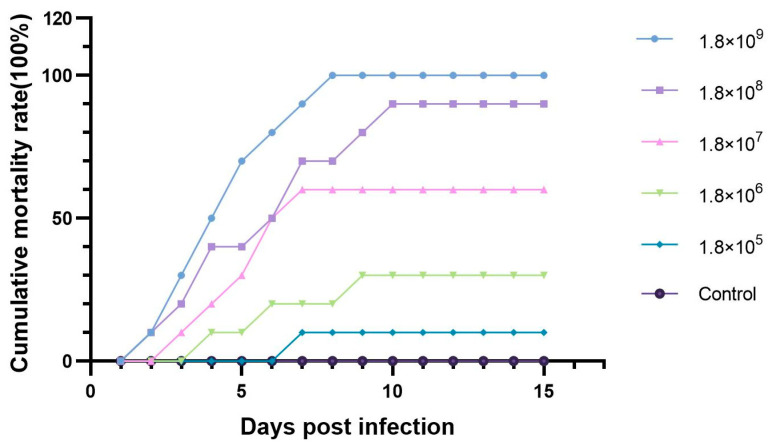
Cumulative mortality of healthy hybrid sturgeons challenged with ST-1902 by intraperitoneal injection. The dose of fish was as follows: 1.8 × 10^9^ CFU/IND, 1.8 × 10^8^ (CFU/IND), 1.8 × 10^7^ CFU/IND, 1.8 × 10^6^ CFU/IND, E: 1.8 × 105 CFU/IND, and control.

In the first 24 h after the challenge, no mortality among the hybrid sturgeons was observed. Mortality occurred gradually in the respective challenge aquaria over a period of 2–8 days. Conversely, no mortality or morbidity was detected in the control group during the three-week challenge period. The cumulative mortalities in the five challenged groups were recorded (Figure 2), and all deceased or moribund fish were collected for bacterial isolation. The median lethal dose (LD_50_) was calculated as 7.9 × 10^6^ CFU/IND using the Spearman–Karber method [28]. The challenged sturgeons exhibited clinical signs similar to those of naturally infected sturgeons, and bacteria were re-isolated from dying sturgeons and re-identified as *A. hydrophila*.

### 3.3. Physiological and Biochemical Characterization of ST-1902

The isolate ST-1902 was motile, Gram-negative rods that were oxidase- and catalase-positive. It fermented and oxidized glucose with gas production. Additionally, ST-1902 was also positive for citrate utilization, lysine decarboxylase production, arginine dehydrogenase activity, H_2_S production, nitrate reduction to nitrite, nitrite reduction to nitrogen, and growth in KCN. However, it was unable to utilize inositol, amygdalin, or galactoside, produce ornithine decarboxylase or phenylalanine deaminase, or hydrolyze urea. Its biochemical profile showed 93.8% similarity to ATCC 7966 (Table 2), the standard isolate of *Aeromonas hydrophila*.

### 3.4. Molecular Identification and Phylogenetic Analysis

**Figure 3:**
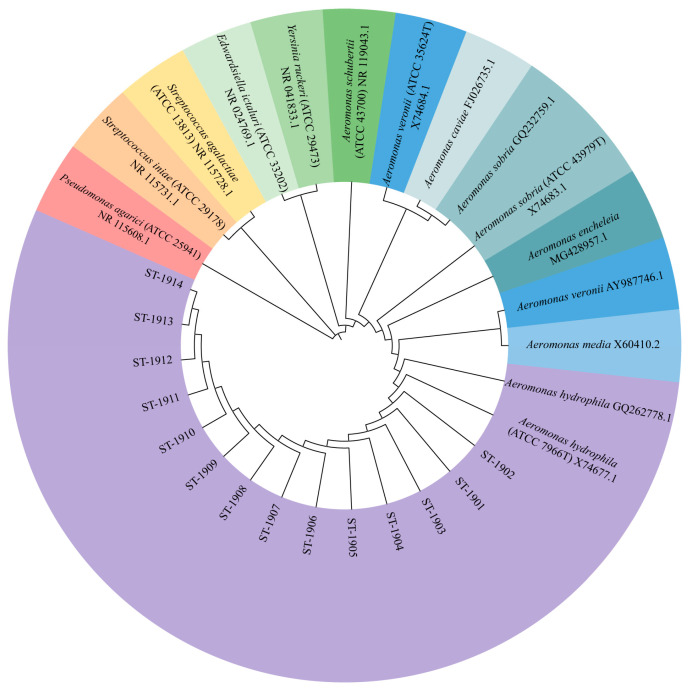
Phylogenetic tree constructed from 16S rDNA sequence analysis of the *A. hydrophia* ST-1902 and other Gram-negative bacteria. The trees were constructed and analyzed by the neighbor-joining methods using 1000 bootstrap replicates. Numbers at the nodes indicate the percentages of reliability of each branch of the tree. Branch lengths are drawn proportional to the estimated sequence divergence.

The 16S rDNA of ST-1902 and the other thirteen strains was amplified, and PCR products measuring 1 450 bp in length were sequenced and registered into the NCBI GenBank database (16S rDNA of ST-1902: MT505702). Subsequently, the 16S rDNA sequences of ST-1902 and other thirteen strains were analyzed by BLAST homology sequence alignment, showing that ST-1902 and the other thirteen strains shared 99% identity with *A. hydrophia* strains. Furthermore, the NJ phylogenetic tree constructed based on 16S rDNA sequences revealed that 14 bacterial strains (indicated by purple markers) clustered into a primary clade with *A. hydrophila*, while showing clear separation from other species of *Aeromonas*, as well as from strains of *Yersinia*, *Edwardsiella*, *Pseudomonas*, and *Streptococcus* (Figure 3). The results of BLAST and phylogenetic analysis confirmed that ST-1902 and the thirteen other isolates belong to the *A. hydrophila* type associated with strains isolated from sturgeons.

### 3.5. Main Virulence Genes for ST-1902

**Figure 4:**
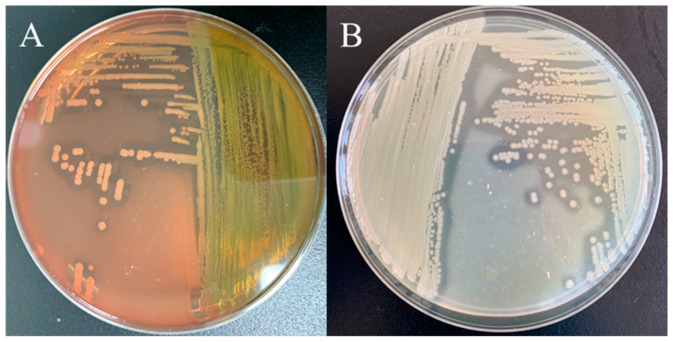
(**A**) The β-hemolysis zone of strain ST-1902 on sheep blood agar plate; (**B**) the transparent proteolytic zone of strain ST-1902 on milk agar.

**Figure 5:**
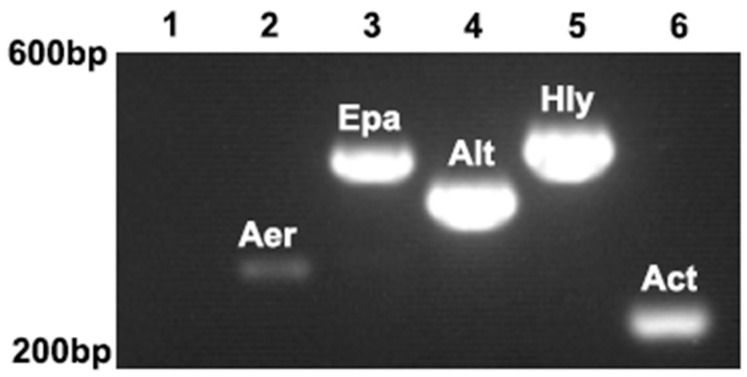
Detection of ST-1902 virulence genes with PCR.

ST-1902 produced hemolysin, as evidenced by a complete β-hemolysis zone around the light-gray, opaque, waxy colonies on sheep blood agar, and a transparent proteolytic ring on milk agar, indicating that ST-1902 can produce hemolysin and extracellular proteases (Figure 4). Using specific primers (Table 1), aerolysin (*Aer*), hemolysin (*Hly*), and extracellular protease (*Epa*) genes were detected with PCR products of 301 bp, 584 bp, and 543 bp, respectively. Additionally, novel enterotoxin genes, including heat-sensitive cytotoxic enterotoxin (*Alt*) and cytotoxic enterotoxin (*Act*), were also detected in ST-1902 (Figure 5). These results corresponded with symptoms of intestinal ulcers, as well as swollen and hemorrhagic anus, observed in both naturally infected and ST-1902-challenged sturgeons (Figure 1B,F).

### 3.6. Histopathological Analysis

**Figure 6:**
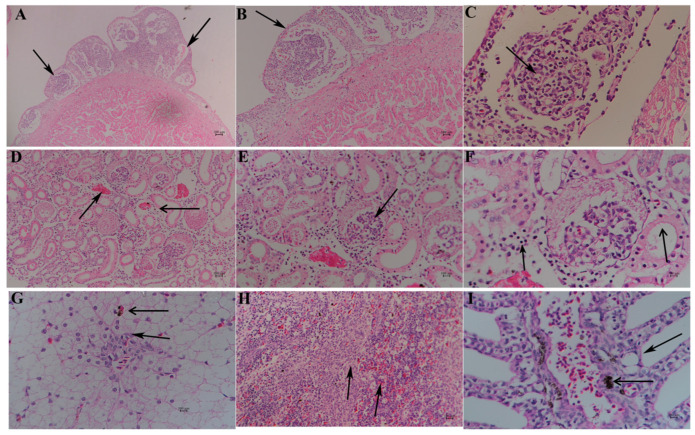
Histopathology of hybrid sturgeon infected with *A. hydrophila* (hematoxylin–eosin staining). (**A**): Hyperplasia on the epicardial mesothelial tissues and cyst cavities (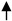) (4×). (**B**): Undifferentiated spherical, spindle cells, and vascular tissue (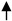) (20×). (**C**): Mononuclear cell (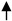) (40×). (**D**): Enlarged renal interstitial space (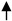) with hyperemia (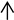) (10×). (**E**): Atrophic glomeruli with inflammatory cell infiltration (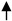) (20×). (**F**): Leucocytes, neutrophils around renal interstitial space (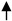), irregular and disassembled nuclei in renal tubule (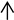) (20×). (**G**): Swollen, vacuolar, and necrotic hepatocytes (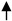), with inflammatory cells around the central vein and hepatic sinus (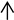) (10×). (**H**): The blurred boundary between the red pulp and white pulp (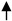) (10×). (**I**): Cavitated gill filaments (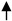) and hemosiderosis on epithelial cells (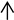).

Sections of neutral formalin-fixed heart, kidney, spleen, liver, and gill from moribund sturgeon were used for staining with hematoxylin–eosin. Obvious hyperplasia and neoplasm-like changes on the epicardial mesothelial tissues can be observed from the heart of diseased sturgeon (Figure 6A), which were easily visible to the naked eye (Figure 1E). The cyst walls, possibly formed by hyperplasia cuboidal epithelium, became thicker with the proliferation and differentiation of cyst. Several undifferentiated spherical and spindle cells, vascular tissue, erythrocytes at different stages of maturity, and inflammatory cells (Figure 6B,C) can be observed within the cyst cavity, indicating inflammatory infiltration. The intercellular space of the kidney became enlarged with necrosis in intercellular tissue and blurred granular boundaries (Figure 6D). A large amount of inflammatory cell infiltration mainly consisting of leucocytes and neutrophils can be observed in renal tubules and slightly atrophic glomeruli, with irregular and disassembled epithelial nuclei in the renal tubules (Figure 6E,F). Swollen, vacuolar, denatured, and necrotic hepatocytes can be observed with many inflammatory cells, mainly consisting of monocytes and other leucocytes around the central vein and hepatic sinus (Figure 6G). Many lymphocytes can be found in the spleen. Blurry boundaries between the red pulp and white pulp were observed with serious hemosiderosis caused by iron overload and capillary bleeding (Figure 6H). The gill filaments were cavitated, and hemosiderosis could be observed in epithelial cells with disintegrated nuclei, which was caused by recurrent bleeding followed by hemosiderin sedimentation.

### 3.7. Antimicrobial Sensitivity and Resistance Gene Analysis

**Figure 7:**
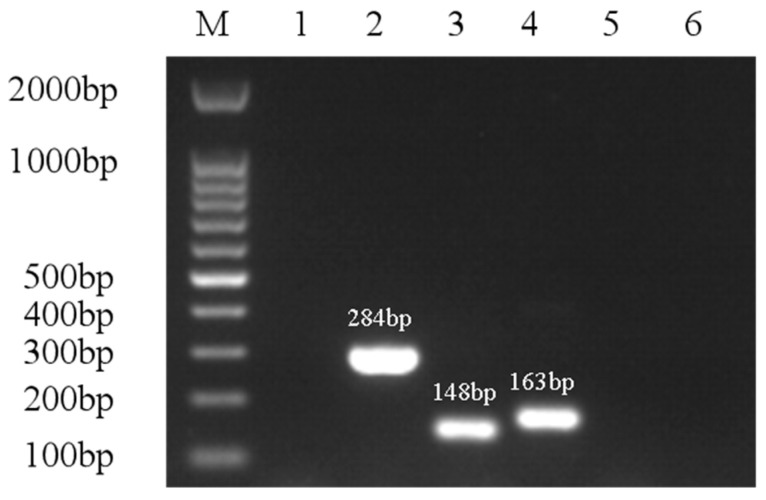
Antimicrobial drug-resistant genes detection with PCR amplification. M, 100 bp DNA Ladder; 1, Negative control; 2, *Ant* (3″)-I gene; 3, *Intl1* gene; 4, *Sul1* gene; 5, *FexA* gene; 6, *Cfr* gene.

The *A. hydrophila* isolate ST-1902 was highly sensitive to ceftriaxone, florfenicol, chloramphenicol, enrofloxacin, and levofloxacin, moderately sensitive to doxycycline, rifampicin, and azithromycin, but resistant to amoxicillin, sulfamethoxazole, gentamycin, tobramycin, kanamycin, neomycin, acetylspiramycin, and vancomycin (Table 3). Resistance gene analysis showed that the ST-1902 strain’s *Ant* (*3”*)-I, *Intl1*, and *Sul1* genes were positive, and the products were 284 bp, 146 bp, and 163 bp, respectively. The *Cfr* and *FexA* genes were negative (Figure 7), which corresponds to the results that ST-1902 was sensitive to non-nicol antibiotics such as florfenicol and chloramphenicol.

## 4. Discussion

Over the past decades, members of *Aeromonas* spp. have frequently been reported as significant pathogens in lower vertebrates, including fishes, amphibians, and reptiles, and are known to induce hemorrhagic septicemia in a wide variety of aquatic animal species [13]. *Aeromonas hydrophila*, a group of Gram-negative short-rod-shaped bacteria (0.3~1.0 × 1.0~3.5 µm), is becoming a major pathogen of cultured sturgeon species, and the mortality even reached 100% [8].

With the worldwide increase in demand for caviar, the sturgeon industry has been expanding in China over the past two decades, particularly focusing on the large hybrid (*Huso dauricus* ♀ *× A. schrenckii* ♂) due to its rapid growth and strong disease resistance [3,5,6]. However, outbreaks of bacterial diseases have surged [7], causing high mortality rates and significant economic losses in sturgeon farms. *Aeromonas hydrophila* and *A. veronii*, the most common aeromonads affecting sturgeon culture, have been reported throughout China and European countries, causing significant economic losses and societal impacts [29,30]. In this study, ST-1902, a typical colony of predominant bacteria isolated from moribund large hybrid sturgeon with hemorrhagic lesions on the skin and anus, was identified as *A. hydrophila*. It was found to be pathogenic with an LD_50_ of 7.9 × 10^6^ CFU/individual for healthy sturgeon with an average weight of 100 g/per fish. ST-1902 exhibited β-hemolysis on sheep blood agar and formed a large transparent zone on milk agar. Detection of virulence genes showed positive results for *Aer*, *Hly*, *Epa*, *Alt*, and *Act*, correlating with symptoms such as intestinal ulceration, as well as swollen and hemorrhagic anus, intestine, spleen, and gonad in infected and challenged sturgeons. This aligns with other research on *A. hydrophila* in sturgeon [9,10,11], indicating the possible virulence mechanisms of ST-1902. Key virulence genes, such as hemolysin (*Hly*), aerolysin (*Aer*), extracellular proteases (*Epa*), and cytotoxic enterotoxins (*Act*, *Alt*), have been identified as critical indicators for determining the virulence of *A. hydrophila*. ST-1902 owns multiple virulence factors, and its interactions with toxins and enzymes enhance the understanding of bacterial pathogenesis [31,32,33,34]. These results suggest that isolate ST-1902 is a major pathogen with high pathogenicity.

Macroscopic symptoms and histopathological changes in the heart, liver, spleen, kidney, and gill were observed using H&E staining. The diseased sturgeon exhibited a mulberry-like lesion on the heart, with neoplasm-like hyperplasia on its surface (Figure 6A). The cyst walls showed thickening due to the proliferation and differentiation of cyst cells, with undifferentiated spherical and spindle cells, vascular tissue, erythrocytes, and inflammatory cells present within the cyst cavity (Figure 6C), indicating inflammation-induced infiltration. The mulberry-like heart is commonly found in hybrid sturgeon cultures in China, with spontaneous steatites on epicardial tissue. Similar symptoms have been observed in cultured Siberian sturgeon infected with *A*. *veronii* and hybrid sturgeon with isolated bacterial pathogens [35,36]. Experimental data suggest that mammalian mulberry-like lesions are associated with deficiencies in trace elements and vitamins such as selenium (Se) and Vitamin E (VE). In addition, some scholars believe that the mulberry-like appearance may be related to high fat content in the fodder, though no relevant proof exists [37,38]. The causes for the mulberry-like heart of cultured sturgeon still require further investigation.

Compared with the increasing resistance of bacteria to antibiotics in commonly cultured fish, caused by the abuse of antimicrobial drugs, *Aeromonas* spp. from sturgeon culture showed less resistance due to effective surveillance by administration offices. This ensures better confirmation of sturgeon and caviar production for increased exportation. The aeromonad isolates from sturgeons showed sensitivity to cefoxitin, kanamycin, gentamicin, doxycycline, and fluoroquinolones [10,39]. In the present study, the drug sensitivity of ST-1902 was analyzed against common antimicrobial drugs (Table 2), showing high sensitivity to ceftriaxone, florfenicol, chloramphenicol, enrofloxacin, and levofloxacin, corresponding to the negative results for the *Cfr* and *FexA* genes. However, ST-1902 is resistant to sulfonamides and four aminoglycoside antibiotics, with positive results for the *Intl1*, *Sul1*, and *Ant* (*3”*)*-I* genes. Sulfonamides were commonly used antimicrobial drugs from 1990 to 2005, leading to many resistant bacterial strains [40]. Greater concern has been raised about aminoglycoside resistance and the detection of the *Ant* (*3”*)*-I* gene, since aminoglycosides were prohibited for use in aquaculture in China. The sources for aminoglycoside-resistant genes require further phylogenetic analysis of the *Ant* (*3”*)*-I* gene with other bacterial strains from clinical and environmental samples. A regular and systematic surveillance system should be established to detect multidrug resistance (MDR) and virulence genes using more cost-effective molecular detection methods to ensure better monitoring and safety in sturgeon culture and global trade.

## Figures and Tables

**Table 1:** Primers used for the detection of virulence genes and antibiotic resistance genes.

Genes	Primer Sequences (5′→3′)	Amplicon Size/bp	Annealing Temperature/°C	GenBank Accession No.
*Aer*	F: CACAGCCAATATGTCGGTGAAG	301	52	MH607886.1
	R: GTCACCTTCTCGCTCCAGGC			
*Hly*	F: GGCCGGTGGCCCGAAGATAGGG	584	64	KP942382.1
	R: GGCGGCGCCGGACGAGACGGG			
*Alt*	F: ATGACCCAGTCCTGGCACGG	482	58	KC695750.1
	R: GCCGCTCAGGGCGAAGCCGC			
*Act*	F: TACCACCACCTCCCTGTCGC	249	60	KC687134.1
	R: ATGCTGCTCGCCTTGTGGTT			
*Epa*	F: TGGTTGTCGGCGTTGTTGAG	543	61	DQ137138.1
	R: TGTGGGTGGACGGAGTGAGT			
*Sul*1	F: CGCACCGGAAACATCGCTGCAC	163	56.3	JN088221
	R: TGAAGTTCCGCCGCAAGGCTCG			
*Intl*1	F: GGCTTCGTGATGCCTGCTT	146	63	MF769724.1
	R: CATTCCTGGCCGTGGTTCT			
*Cfr*	F: TGTGCTACAGGCAACATTGGAT	148	55	AJ249217
	R: CAAATACTTGACGGTTGGCTAGAG			
*Ant* (3″)-I	F: TGATTTGCTGGTTACGGTGAC	284	53	FJ644661.1
	R: CGCTATGTTCTCTTGCTTTTG			
*FexA*	F: ATTCTCCCGCAAATAACG	156	52	AJ549214
	R: TCGGCTCAGTAGCATCACG			

**Table 2:** Biochemical characteristics of strain ST-1902 and ATCC 7966.

Biochemical Items	ST-1902	ATCC 7966	Biochemical Items	ST-1902	ATCC 7966
Gram stain	−	−	Methyl red	+	+
Oxidase	+	+	Glucose	+	+
Catalase	+	+	Mannitol	+	+
Motility	+	+	Inositol	−	−
Glucose fermented	+	+	Sorbitol	+	−
Galactoside	−	−	Rhamnol	+	D
Arginine	+	+	Sucrase	+	+
Lysine	+	+	Melibiose	−	−
Ornithine	−	−	Amygdalin	−	−
Phenylalanine TDA	−	D	Arabinose	+	+
Citrate	+	D	Nitrate reduction	+	+
Hydrothion	+	+	Nitrite reduction	+	+
Urea	−	−	H_2_S	+	+
Indole	+	+	KCN	−	−
Pyruvate	+	D	SS Agar	+	+
Gelatin	+	+	MacConkey Agar	+	+

Note: “+”, positive; “−”, negative.; “D”, different.

**Table 3:** Antimicrobial susceptibility test to ST-1902.

Drugs	IZD/mm	Resistance	Drugs	IZD/mm	Resistance
Sulfamethoxazole	8	R	Enrofloxacin	24	S
Amoxicillin	8	R	Levofloxacin	22	S
Ceftriaxone	32	S	Chloramphenicol	22	S
Vancomycin	0	R	Florfenicol	24	S
Neomycin	6	R	Azithromycin	14	I
Gentamicin	10	R	Acetylspiramycin	4	R
Kanamycin	8	R	Rifampicin	18	I
Tobramycin	10	R	Polymyxin B	6	R
Amikacin	9	R	Doxycycline	18	I

## Data Availability

The original contributions presented in the study are included in the article, further inquiries can be directed to the corresponding authors.

## Appendix B

**Table A1:** Information of isolated strains and sources.

	Tissue					
Date	Liver	Spleen	Kidney	Ascites	Total	Number of the Strains
1 July 2019	1	1	1	4	7	ST-1901~ST-1907
5 July 2019	1	1	1	2	5	ST-1908~ST-1912
10 July 2019	1	0	0	1	2	ST-1913~ST-1914
